# Tumour-infiltrating lymphocytes and response to neoadjuvant letrozole in patients with early oestrogen receptor-positive breast cancer: analysis from a nationwide phase II DBCG trial

**DOI:** 10.1186/s13058-020-01285-8

**Published:** 2020-05-14

**Authors:** Signe Korsgaard Skriver, Maj-Britt Jensen, Ann Soegaard Knoop, Bent Ejlertsen, Anne-Vibeke Laenkholm

**Affiliations:** 1 Department of Oncology, Rigshospitalet, Copenhagen University Hospital, section 5703 Rigshospitalet, Blegdamsvej 9, DK-2100 Copenhagen, Denmark; 2 The Danish Breast Cancer Group, Rigshospitalet, Copenhagen University Hospital, Copenhagen, Denmark; 3 Department of Surgical Pathology, Zealand University Hospital, Roskilde, Denmark

**Keywords:** Breast neoplasms, Neoadjuvant, Endocrine therapy, Letrozole, TILs

## Abstract

**Background:**

The presence of tumour-infiltrating lymphocytes (TILs) is associated with response to neoadjuvant chemotherapy among patients with triple-negative and HER2-positive breast cancer. However, the significance of TILs is less clear in luminal breast cancer. Here, we in postmenopausal patients with primary oestrogen receptor-positive (ER+), HER2 normal, operable breast cancer assessed the importance of inducing TILs during 4 months of letrozole on response in a neoadjuvant phase II study.

**Methods:**

Participants were postmenopausal women with ER+, HER2 normal operable breast cancer assigned to 4 months of neoadjuvant letrozole. Pretreatment core biopsies and surgical specimens were assessed centrally for the percentage of TILs on haematoxylin and eosin-stained slides according to the International Immuno-Oncology Biomarker Working Group on Breast Cancer guidelines. Pathological response was assessed by the Residual Cancer Burden (RCB) index and a modified Miller-Payne grading system and was analysed according to change in TILs.

**Results:**

Tumour specimens were available from 106 of the 112 patients treated per protocol. TIL concentration increased with mean 6.8 percentage point (*p* < 0.0001) during treatment (range − 39 to 60). An increase in TILs was significantly associated with pathological response with OR = 0.71 (95% CI 0.53–0.96; *p* = 0.02) per 10% absolute increase for pathological response and correspondingly OR = 0.56 (95% CI 0.40–0.78; *p* = 0.0007) for lower RCB index per 10% increase.

**Conclusion:**

Increasing TILs during letrozole was significantly associated with a poor treatment response. An increase in TILs during endocrine therapy might imply immunogenicity, and these patients could be targetable by immunotherapy.

**Trial registration:**

ClinicalTrials.govNCT00908531, registered 27 May 2009.

## Background

Tumour-infiltrating lymphocytes (TILs) have been established as a predictive biomarker for response to neoadjuvant chemotherapy irrespective of molecular subtype [1]. The evidence is most pronounced in triple-negative and HER2-positive breast cancer, and likewise, increased TILs are a strong prognostic factor for improved survival in early triple-negative and HER2-positive breast cancer [2, 3]. In contrast, the presence of TILs has in oestrogen receptor-positive (ER+) breast cancer been suggested to be an adverse prognostic factor and associated with poor response to aromatase inhibitors [1, 4, 5]. While the composition and dynamics of TILs are complex, several studies have shown that the predictive information from immune gene expression analyses correlates well with TIL count on haematoxylin and eosin-stained slides [6, 7].

Neoadjuvant studies have a major strength that by comparing sequential specimens from patients before and after treatment it is possible to obtain predictive and prognostic information using tumour response, linking biology with clinical response. Response to neoadjuvant treatment can be used to stratify post neoadjuvant treatment. Pathological complete response (pCR) has been the most commonly used endpoint in neoadjuvant trials, but a low pCR rate in ER+ breast cancer makes pCR a less than ideal endpoint in this population. Different pathological scoring systems for residual disease (RD) exist including the Miller-Payne grading system, which estimates a decrease in cellularity during treatment [8], and the Residual Cancer Burden (RCB) index that take tumour dimensions, cellularity of the tumour bed and axillary nodal burden into account [9, 10]. For neoadjuvant endocrine treatment, the Preoperative Endocrine Therapy Prognostic Index (PEPI) score has been applied in some occasions [11]. The PEPI score is however not routinely used mainly due to the lack of standardisation and validity of Ki67 scoring [12].

In this study, we investigated the dynamics of TILs and relationship to pathological outcome during neoadjuvant endocrine therapy.

## Methods

### Study population

Patients were treated with neoadjuvant letrozole for 4 months prior to curative intended surgery as part of a clinical phase II study conducted by the Danish Breast Cancer Group (DBCG) between 2009 and 2012. The study is registered on clinicaltrials.gov (NCT00908531). The original study design and clinical results have been previously published [13]. Endpoints were clinical and pathological outcome. A total of 119 patients were registered to receive neoadjuvant letrozole. Eligible patients had histological confirmed, invasive, ER+, HER2-negative, operable breast cancer. They met the following criteria: tumour size ≥ 1 cm, ≥ 60 years at entry, Eastern Cooperative Oncology Group score 0–2 and Charlson comorbidity index 0–2. Patients with prior cytotoxic treatment including aromatase inhibitors and prior malignant disease were not eligible. Patients were registered in the DBCG database and updated prospectively. Four patients were excluded prior to study initiation, two cases due to HER2 positivity at central testing and two patients withdrew consent. An additional three were excluded from the intention-to-treat population. One hundred six patients had paired tissue samples from biopsy and surgery and were included in the present study (supplementary Fig. 1).

### Biomarker analyses

ER, PGR, HER2 and Ki67 were assessed centrally using international standards [14–16]. ER and Ki67 were recorded as continuous variables. Tumours were considered ER positive when nuclear staining was equal to or higher than 10%. TILs were assessed by the use of the guidelines of the International Immuno-Oncology Biomarker Working Group on Breast Cancer [17]. Pre- and posttreatment stromal TILs were quantified on haematoxylin and eosin-stained slides, from biopsies and surgical specimens respectively, as percentage infiltration of mononuclear cells. TILs were assessed both continuously and categorised into groups: low (0–9%), intermediate (10–59%) and high (60–100%). Changes in TILs were calculated as the difference between pre- and posttreatment TIL counts and categorised as decreased when TILs dropped ≥ 10 percentage point, as increased when TILs elevated ≥ 10 percentage point or as no change (less than 10 percentage point decrease or increase).

### Endpoint

Endpoint was to determine the association between changes in TILs during neoadjuvant endocrine treatment and achieving a pathological response. Pathological response was defined as a decrease in tumour cells ≥ 30% according to a modified Miller-Payne grading scale used by the DBCG [8]. On the modified scale response, grade 1 equals no invasive cells present in the tumour bed, pathological complete response (pCR). Grade 2 more than 90% loss of tumour cells and grade 3 between 30 and 90% reduction in tumour cells were considered partial response. Grade 4 is defined as less than 30% loss of tumour cells and was considered no response. Exploratively pathological response was also assessed on the RCB index scoring RD both continuously and categorically using the guidelines presented by the BIG-NABCG collaboration [9, 10]. In brief, the RCB index combines the bidimensional diameter of the primary tumour with the percentage of invasive cells in the tumour, corrected for the percentages of in situ carcinoma, and the number of positive lymph nodes including the diameter of the biggest lymph node metastasis in a generalised linear model. The higher RCB index, and the corresponding RCB-class, the more extensive residual disease load. For the RCB index calculations in this study, we used the online Residual Cancer Burden Calculator provided by the MD Andersson Cancer Center [18]. As an additional exploratory analysis, we assessed response after the PEPI scoring system. The PEPI score combines pathological tumour size, ER, Ki67 and node status in a score from 0 to 12 where a low PEPI score indicates excellent prognosis.

### Statistical methods

The distribution of TILs in standard clinicopathological subgroups was tested with chi-square or Fisher’s exact test. The distribution of changes in TILs did not meet the assumption of normality, and the Wilcoxon signed rank test was used to test for changes in distribution pre- and posttreatment. The association with pathological outcome was tested with univariate logistic regression. Factors were included in univariate models both categorically and continuously to investigate the functional form. Unknowns were included in separate categories. Multivariate analyses including factors that were statistically significant in the univariate analyses were applied to assess the adjusted odds ratios. Factors that were not statistically significant in the multivariate analysis were excluded from the final model. Odds ratio (OR) was estimated with a 95% confidence interval (CI), using the category with the highest number of patients as the reference group, except for PGR to align it with ER. The exploratory variables RCB and PEPI score were only tested in univariate analysis. Correlation between TILs and other biomarkers was tested with Pearson’s correlation. The level of significance was set to 5%. All *p* values are two-sided. All analyses were performed with SAS enterprise guide version 7.15 (Cary, NC, USA).

## Results

### Patient demographics and pretreatment TILs

Patients’ basic characteristics are summarised in Table 1. Median age was 67 years (range 60–87 years). All patients had pretreatment TIL assessment, with a baseline median of 5%, range 0–50%. Apart from Ki67, there were no statistically significant differences between patient with low and intermediate levels of pretreatment TILs in the different clinicopathological subgroups (Table 1).

**Table 1:** Patient and tumour characteristics of the study population

	All patients, *n* = 106		Patients with low TILs, *n* = 81		Patients with intermediate TILs, *n* = 25		
Characteristic	*n*	% (col)	*n*	% (col)	*n*	% (col)	*p*^1^
Age (years)							0.12
60–69	44	(41)	44	(54)	18	(72)	
70–89	62	(59)	37	(46)	7	(28)	
Tumour size (mm)^2^							0.32
< 20	46	(43)	33	(41)	13	(52)	
≥ 20	60	(57)	48	(59)	12	(48)	
Histological subtype							0.40
Ductal	76	(72)	56	(70)	19	(76)	
Lobular	12	(11)	11	(14)	1	(4)	
Other invasive^3^	17	(16)	13	(16)	5	(20)	
Unknown	1	(1)	1	(1)	0		
Malignancy grade^4^							0.96
1	37	(43)	28	(42)	9	(45)	
2	45	(52)	35	(52)	10	(50)	
3	5	(6)	4	(6)	1	(5)	
Axillary node status							0.08
Negative	58	(55)	41	(51)	17	(68)	
Positive	47	(44)	40	(49)	7	(28)	
Unknown	1	(1)	0		1	(4)	
Oestrogen receptor status (%)							0.18
10–99	20	(19)	13	(16)	7	(28)	
100	86	(81)	68	(84)	18	(72)	
Progesterone receptor status (%)							0.20
10–99	75	(71)	57	(70)	18	(72)	
100	29	(27)	22	(27)	7	(28)	
Unknown	2	(2)	2	(3)	0	(0)	
Ki67 index (%)							**0.02**
< 14	72	(68)	60	(74)	12	(48)	
≥ 14	32	(30)	20	(25)	12	(48)	
Unknown	2	(2)	1	(1)	1	(4)	

^1^Excluding unknowns; ^2^range 11–100 mm; ^3^other invasive: mucinous carcinomas *n* = 8, tubular carcinomas *n* = 2, medullary carcinoma *n* = 1, not specified *n* = 6; ^4^only lobular and ductal tumours graded, *n* = 88

*TILs*, tumour-infiltrating lymphocytes

### Pathological response

Assessment of pathological response according to Miller-Payne was available for 102 patients (96%). Of them, 58 (57%) had pathological response including one patient with pCR. Forty-four (43%) had no response. Assessment of RCB was possible for all 106 patients: range RCB index 0–3.649, and when grouped into RCB-classes: one patient had RCB-class 0 (pCR), six (6%) had RCB-class I, 90 (83%) had RCB-class II and 12 (11%) had RCB-class III. Assessment of the PEPI score was possible for 104 patients, with 45 (43%) patients obtaining a PEPI score of 0 and 59 (57%) a PEPI score between 1 and 6 (mean score 1.7) (supplementary Table 1).

### TIL changes during treatment

TIL concentration increased overall with a mean of 6.8 (*p* < 0.0001) during treatment (range − 39 to 60). Thirty-nine (37%) patients had an increase in TIL concentration of 10% or higher (mean 23%, range 10–60%), 12 (11%) had a decrease in TIL concentration (mean drop 19%, range 10–39%) and the remaining 55 patients had stable TILs.

**Fig. 1:**
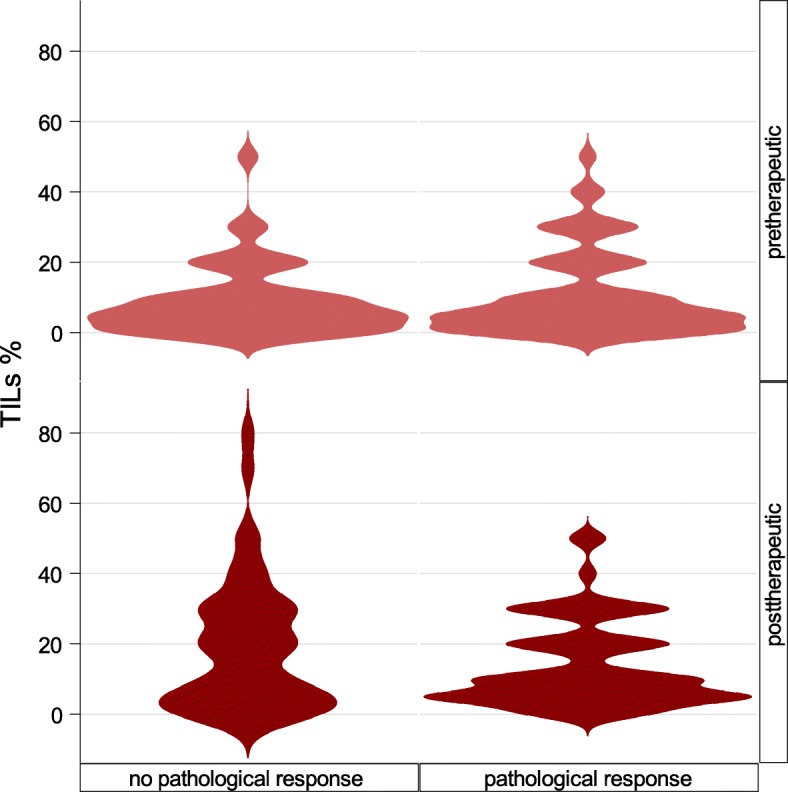
Bean plot illustrating distributions of TILs before and after neoadjuvant endocrine treatment in patients with no pathological response and in patients with pathological response (complete or partial). Pre- and posttreatment distribution differs significantly in patients with no pathological response (*p* ≤ 0.0001), but not in patients with response (*p* = 0.1)

When divided into responders and non-responders, the increase in concentration was significant among non-responders with a mean increase of 10.6 (*p* < 0.0001) compared to responders (mean increase 3.2, *p* = 0.10); these data are presented in Table 2 and Fig. 1.

**Table 2:** TIL increase during neoadjuvant letrozole in postmenopausal breast cancer patients (*n* = 106)

	Change	*p*
Overall increase, mean (range)	6.8 (− 39 to 60)	**< 0.0001**
Responders (*n* = 58)	3.2 (− 39 to 35)	0.10
Non-responders (*n* = 44)	10.6 (− 25 to 60)	**< 0.0001**

*TILs* tumour-infiltrating lymphocytes

### Variables’ relation to outcome

All clinicopathological factors listed in Table 1 are tested for association to pathological response. Tumour size below 2 cm, a ductal subtype, low posttreatment Ki67 and stable or decreasing TILs were predictive of pathological response in the univariate analysis. When tested in multivariable analyses, only TILs and subtype remained significant for pathological response. In the final model, OR estimates for pathological response were 0.71 (95% CI 0.53–0.96; *p* = 0.02) per 10% absolute increase in TILs and 0.37 (95% CI 0.15–0.91; *p* = 0.03) for non-ductal subtype as presented in Table 3. For the explorative endpoint, RCB TILs were significantly predictive for response with OR = 0.56 (95% CI 0.40–0.78; *p* 0.0007) per 10% absolute increase in TILs and posttreatment Ki67 was borderline significant with OR 0.77 (CI 0.60–1.00; *p* = 0.05) per 10% increase in Ki67 index. There was no significant association between TILs and achievement of a PEPI score of 0: OR 0.85 (CI 0.66–1.10; *p* = 0.22) per 10% increase in TILs.

**Table 3:** Univariate and multivariate analyses for pathological response in patients treated with neoadjuvant endocrine letrozole

Variable^1^	Pathological response, *n* (102)		Odds ratio (95% CI) univariate	*p*^2^	Odds ratio (95% CI) multivariate	*p*^2^
	Yes	No				
TILs (%)^3^ pretreatment			1.20 (0.84–1.72)	0.31		
TILs (%)^3^ posttreatment			0.78 (0.60–1.02)	0.07		
TIL change (%)^3^			0.71 (0.53–0.96)	**0.02**	0.71 (0.53–0.96)	**0.02**
KI67 index (%)^3^ pretreatment			0.99 (0.96–1.01)	0.22		
Ki67 index (%)^3^ posttreatment			0.53 (0.31–0.91)	**0.02**		
Age (years)						
60–69	34	28	0.81 (0.36–1.81)	0.61		
70–89	24	16	1			
Tumour size (mm)				**0.03**		
< 20	31	14	2.46 (1.10–5.57)			
≥ 20	27	30	1			
Subtype				**0.03**		**0.03**
Ductal	46	26	1		1	
Other invasive	12	18	0.38 (0.16–0.90)		0.37 (0.15–0.91)	
Malignancy grade				0.26		
1	22	13	1.07 (0.42–2.66)			
2	27	17	1			
3	1	4	0.16 (0.02–1.53)			
Axillary node status prior to treatment				0.99		
Negative	31	24	1			
Positive	26	20	1.01 (0.46–2.22)			
Unknown	1	0	–			
Oestrogen receptor status (%)				0.15		
10–99	8	11	0.48 (0.18–1.32)			
100	50	33	1			
Progesterone receptor status (%)				0.78		
0–99	41	32	0.88 (0.36–2.16)			
100	16	11	1			
Unknown	1	1	–			

Data reported as *n*. ^1^Unless otherwise specified, variables are pretreatment values. ^2^Excluding unknowns. ^3^Continuous variable, per 10% increase

*TILs*, tumour-infiltrating lymphocytes

### Correlation between TILs and standard pathological variables

Ki67 levels paralleled pretreatment TIL levels. The correlation between pretreatment TILs and Ki67 was moderate (Pearson 0.4; *p* = 0.0002); however, the association grew stronger posttreatment (Pearson 0.5; *p* < 0.0001). TILs were weakly correlated to ER (Pearson − 0.2; *p* = 0.02 pretreatment and − 0.3; *p* = 0.0006 posttreatment) and not significantly correlated to PgR (Pearson − 0.04; *p* = 0.70 pretreatment and − 0.2; *p* = 0.21 posttreatment).

## Discussion

We found that an increase in TILs during neoadjuvant letrozole was associated with a poor treatment response, regardless of the pathological assessment method. Furthermore, TILs were positively correlated with Ki67 levels both before and after 4 months of letrozole. A high Ki67 index is indicative for a luminal B subtype, which harbours a greater genomic instability than luminal A [19]. A high number of mutations increase the chance that mutated protein sequences will be exposed and recognised as neo-antigens by the immune system, hereby activating the immune system and promoting lymphocytic infiltration of the tumour area. The relationship between Ki67 and TILs in ER+, HER2 normal breast cancer has been examined by others with differing results. Denkert et al. [2] showed that stratifying ER+, HER2 normal breast cancers in high/low Ki67 did not change the overall prognostic effect of TILs, whereas Fujimoto et al. [20] demonstrated that high TILs were associated with favourable DFS in Ki67-high, but not in Ki67-low ER+, HER2 normal breast cancer. As more data will become available, we will gain a deeper understanding of the interaction between the tumour, its microenvironment and the adaptive immune activation system and the implications for prognosis and treatment efficacy.

Our data confirms previous finding by our group and others that tumours of non-ductal histologic subtype predict a poor response to neoadjuvant therapy [13, 21, 22].

Contradictory results regarding TILs were found by Liang et al. in the CARMINA 02 trial [23]. When assessing 83 patients treated with endocrine treatment, they found TILs increased in responders but remained stable in non-responders. As in our study, pretreatment levels of TILs did not predict response. The dissimilarities in our results call for testing in larger studies and alignment of pathological assessment method used. Baseline TILs in our population ranged between 0 and 50%, which is consistent with the data from Stanton et al. [24] showing that 94% of ER+ menopausal breast cancer patients had less than 49% TILs when diagnosed (*n* = 2410). Our data supports that of others that ER-positive tumours with higher lymphocytic infiltration have reduced benefit from aromatase inhibitor treatment [4].

The current study has some limitations. Firstly, the small sample size in the study results in a limited power, and secondly, we do not have knowledge of the composition of immune cells beyond TIL count in our population. The strengths of our study include prospectively planned diagnostic and pathological procedures uniformly carried out with central assessment after international recommended guidelines. Patient treatment and follow-up are performed according to DBCG national guidelines [25]. A general limitation for neoadjuvant endocrine studies is the lack of validated methods for response evaluation. The Miller-Payne grading system and the RCB index is both developed and validated in neoadjuvant studies for chemotherapy and does not necessarily translate into the neoadjuvant endocrine setting. The PEPI score, which has been applied in the neoadjuvant endocrine setting, lacks analytical validity across laboratories rendering it, so far, only feasible for research [12]. The results from this study indicate that RCB might be used for the measurement of neoadjuvant endocrine treatment response since an increase in TILs during treatment was predictive for a poor response, irrespective of the applied response evaluation method.

More knowledge is needed to predict response to endocrine treatment based on the dynamic and composition of TILs, especially more detailed knowledge on the composition of immune cells in luminal breast cancer. Previous studies on TILs in breast cancer identified lymphocyte populations that were mainly comprised of CD8+ cytotoxic T cells and CD4+ regulatory cells together with varying proportion of other helper T cells, B cells and NK cells [26]. CD8+ is the key immune cell for tumour cell elimination, working partly through cytotoxic granule release mediated by Granzyme B (GrB) [24, 27]. The only known human intracellular inhibitor of GrB is serine proteinase inhibitor 9 (PI-9). In vitro studies have revealed that elevated levels of oestrogen receptor alpha induce PI-9 [27]. In postmenopausal women, circulating oestrogen is on average 50 pM, which is sufficient to induce PI9 [27, 28]. Another study investigating lymphocyte composition changes during neoadjuvant endocrine treatment found that oestrogen depletion resulted in a significant increase of the CD8+/Treg ratio [29].

Breast cancer in general, and especially luminal breast cancer, is considered non-immunogenic, and immunotherapy has yet to have a place in the adjuvant setting for breast cancer patients. TIL count and modulation of immune response could be used to select patients for inclusion in adjuvant immunotherapy trials. Additionally, it is rational to enhance the anti-tumour immune response prior to potential immunotargeted treatment. CDK4/6 inhibitors promote the recruitment of TILs, as a result of several mechanisms including enhanced tumour antigen presentation, reduced proliferation of immunosuppressive regulatory T cells and a direct stimulatory effect on T cells [30, 31]. Although these results are from preclinical studies, the possibilities are promising and possible combinations with targeted and immunotherapy are currently being investigated both in laboratories and in clinical trials.

## Conclusions

Increasing TILs during neoadjuvant endocrine therapy is predictive for a poor pathological response. These patients could be candidates for immunotherapy, but more research on the subject is needed.

## Supplementary information

**Additional file 1: Supplementary Fig. 1.** Flow diagram of the study population. **Supplementary table.** Distribution of PEPI score in patients treated with neoadjuvant letrozole between 2009 and 2012.

## Data Availability

The data supporting all the figures, tables and supplementary tables in the published article are not publicly available due to institutional restrictions. The dataset can be made available to qualified researchers through application to the Danish Breast Cancer Group. Please contact dbcg.rigshospitalet@regionh.dk.

## Abbreviations

BIG-NABCG: Breast International Group-North American Breast Cancer Group
CI: Confidence interval
DBCG: Danish Breast Cancer Group
ER+: Oestrogen receptor positive
HER2: Human epidermal growth factor receptor 2
IHC: Immunohistochemistry
OR: Odds ratio
pCR: Pathological complete response
PEPI: Preoperative Endocrine Therapy Prognostic Index
PGR: Progesterone receptor
RCB: Residual Cancer Burden
RD: Residual disease
TILs: Tumour-infiltrating lymphocytes
