# Supplementary material for: Tumour-infiltrating lymphocytes and response to neoadjuvant letrozole in patients with early oestrogen receptor-positive breast cancer: analysis from a nationwide phase II DBCG trial
Source: Breast Cancer Res. 2020 May 14;22:46. doi: 10.1186/s13058-020-01285-8 (PMC7222485; doi:10.1186/s13058-020-01285-8)
Supplement: Supplementary file 1 — Additional file 1: Supplementary Fig. 1. Flow diagram of the study population. Supplementary table. Distribution of PEPI score in patients treated with neoadjuvant letrozole between 2009 and 2012. [file 13058_2020_1285_MOESM1_ESM.docx]

**Supplementary figure A**. Flow diagram of the study population

Included

(*n* = 119)

Excluded prior to treatment

- HER2 positive (*n* = 2)
- Withdrew consent (*n* = 2)

Intention to Treat

(*n* = 115)

Excluded after treatment initiation

- HER2 positive (*n* = 2)
- Diagnosed with other malignancy in the trial period (*n* = 1)

Per protocol analyses

(*n* = 112)

Paired tissue samples

(*n* = 106)

| **Supplementary Table 1.**    Distribution of PEPI score in patients treated with neoadjuvant letrozole between 2009 and 2012. n = 104 | | | | |  |
| --- | --- | --- | --- | --- | --- |
| PEPI score | | n | % |  | |
|  | 0 | 45 | (43) |  | |
|  | 1 | 13 | (13) |  | |
|  | 2 | 5 | (5) |  | |
|  | 3 | 22 | (21) |  | |
|  | 4 | 13 | (13) |  | |
|  | 5 | 3 | (3) |  | |
|  | 6 | 3 | (3) |  | |
| PEPI: Preoperative Endocrine Therapy Prognostic Index | | | | |  |
